# Japan-British Exhibition Awards

**Published:** 1910-09-24

**Authors:** Wm. Planner

**Affiliations:** Secretary


*To the Editor of* THE HOSPITAL.

### JAPAN-BRITISH EXHIBITION AWARDS.

*To the Editor of* THE HOSPITAL.

SIR,—On July 9 the name of our firm appeared in the list of awards as the only recipients of the Grand Prix for disinfectants, and we duly announced that fact by an advertisement in the columns of your journal. We now learn (eight weeks after the original publication of the list), that within the last few days a similar distinction has been conferred upon another firm of manufacturers, making our statement erroneous as at the present time. We now ask the courtesy of your columns to correct our statement, which was, of course, made in good faith, and in no sense intended to mislead.

Yours, etc.,

For Jeyes' Sanitary Compounds Co., Ltd.,

WM. PLANNER (Secretary).

